# Reasonable deep application of sheep manure fertilizer to alleviate soil acidification to improve tea yield and quality

**DOI:** 10.3389/fpls.2023.1179960

**Published:** 2023-06-23

**Authors:** Xiaoli Jia, Yuhua Wang, Qi Zhang, Shaoxiong Lin, Ying Zhang, Mengru Du, Meihui Chen, Jianghua Ye, Zeyan Wu, Haibin Wang

**Affiliations:** ^1^ College of Tea and Food, Wuyi University, Wuyishan, China; ^2^ College of Life Science, Fujian Agriculture and Forestry University, Fuzhou, China; ^3^ College of Life Science, Longyan University, Longyan, China

**Keywords:** sheep manure fertilizer, tea plantation, nitrogen transformation, microorganisms, soil enzymes, gene expression, pH value

## Abstract

Soil acidification in Chinese tea plantations is widespread, and it has significantly affected the growth of tea trees; it was important to explore soil remediation of acidified tea plantations in depth for the sustainable development of tea industry. In this study, the effects of sheep manure fertilizer with different application depths on soil acidification, tea yield and quality, and soil nitrogen transformation in tea plantations were analyzed for five consecutive years from 2018 to 2022. The results showed that long-term use of sheep manure fertilizer significantly reduced soil acidification (*P*< 0.05) in tea plantations, improved soil pH and soil ammonium nitrogen content, enhanced root activity and root nitrogen uptake capacity of tea trees, and thus improved tea yield and quality. The effect of different application depths of sheep manure fertilizer on tea yield and quality was mainly reflected in the transformation ability of soil ammonium nitrogen and nitrate nitrogen, which showed that high transformation ability of soil ammonium nitrogen and high ammonium nitrogen content were beneficial to high tea yield and vice versa, and the best effect was achieved when sheep manure was applied at a depth of 50 cm and 70 cm. The topsis analysis confirmed that sheep manure fertilization had a greater effect on root activity, ammonium nitrogen, ammonia intensity, and *nifH* gene. This study provided an important practical basis for the restoration of acidified tea plantation soil through sheep manure fertilizer management.

## 1 Introduction

Tea tree (*Camellia sinensis*) is an important cash crop that plays an important role in promoting agricultural development. According to the statistics of the International Tea Committee (ITC), in 2023, the world tea planting area reached 5.097 million hectares, an increase of 1.257 million hectares over 2010, with a compound annual growth rate of 2.7%. In 2023, world tea production reached 6.397 million tons, an increase of 1.808 million tons over 2010, with a compound annual growth rate of 3.3%.

Tea trees are distributed throughout the world, are *Camellia sinensis* evergreen shrubs or small trees, leaves thin leathery, elliptic-lanceolate or long elliptic, petiole short, apex obtuse; flowers present in cymes, white, pedicels recurved; capsules appear spherical; seeds are brownish. The tea plantation should have an annual rainfall of more than 1500 mm, an average annual temperature of 18-25°C, sufficient sunlight, an altitude of less than 1000 m, and a soil pH of 4.5-5.5 (Wang et al., 2020). China is the world’s largest tea growing country. As a cash crop, a tea tree is mainly harvested in the form of buds and leaves during its growth, so it has a large demand for fertilizer (Liu et al., 2018). According to the report, the annual use of fertilizer on tea trees was more than twice the recommended amount. Heavy fertilizer use did not effectively ensure tea yield and quality, but aggravated the degree of soil acidification (Akiyama et al., 2006; Ni et al., 2019). In recent years, soil acidification in Chinese tea plantations has been widespread (Yan et al., 2020b). The tea tree was an acid-loving crop and its suitable soil pH for cultivation was between 4.5 and 5.5 (Mohammad et al., 2014; Mehra and Baker, 2017). When soil pH< 4.5, tea tree root growth was inhibited and the number of new roots was significantly reduced, which in turn contributed to a significant decrease in tea tree biomass, etc. (Sun et al., 2020). The destruction of the root system of the tea tree directly affected its nutrient uptake and utilization efficiency, which in turn reduced tea yield and quality (Ding et al., 2022; Wang et al., 2022c). Some studies have shown that the use of rape cake and cow manure fertilizer can effectively alleviate soil acidification and thus improve crop yield and quality (Xie et al., 2021; Le et al., 2022; Yin et al., 2022). Organic fertilizers, usually derived from plants or animals, not only increased soil organic matter content and provided nutrients for crops, but also promoted soil microbial propagation, which in turn improved soil ecology (Chepkorir et al., 2018; Chernov and Semenov, 2021; Prajna et al., 2022). Tea trees are usually harvested or pruned every year, so their growth requires a large amount of soil fertilizers, especially nitrogen fertilizers (Liu et al., 2018). Meanwhile, the tea tree was an ammonium-loving plant, and ammonium nitrogen was beneficial to the root development of the tea tree and improved the accumulation of free amino acids in tea leaves (Li et al., 2020b). Numerous studies showed that soil pH affected soil nitrogen transformation, as shown by a decrease in soil pH, a decrease in ammonium nitrogen content and an increase in nitrate nitrogen content (Stopnisšek et al., 2010; Scarlett et al., 2021). Soil nitrogen transformation and its content were closely related to the number and intensity of related microorganisms and ammonifying bacteria facilitated the transformation of soil organic and mineralized nitrogen into ammonium nitrogen (Bojarszczuk et al., 2019; Yan et al., 2020a), while nitrifying bacteria converted soil ammonium nitrogen into nitrate nitrogen (Wang et al., 2022b; Yu et al., 2022). It can be seen that the application of organic fertilizer could improve soil pH value, improve soil quality, promote microbial propagation related to ammonium nitrogen transformation, increase soil ammonium nitrogen content, and thus improve crop yield and quality (Ye et al., 2022; Ye et al., 2023).

In the early stages, our research team used sheep manure fertilizer, cow manure fertilizer and pig manure fertilizer to treat acidified tea plantation soil, and found that sheep manure fertilizer significantly reduced soil acidification (*P*< 0.05) and had the best effect on improving tea yield and quality (Ding et al., 2018). On the basis of our previous research, we continued to conduct in-depth research on the improvement scheme of sheep manure fertilizer on acidified tea plantation soil. Plants mainly absorb nutrients through the root system, which varies in development and depth of root distribution in the soil layer for different plants (Wu et al., 2022). The root system of adult shrub tea trees was usually between 50 and 80 cm. We can’t modify soil texture, however, soil texture could be improved through sheep manure fertilizer. Sheep manure fertilizers were usually applied by ditching and burying around the roots of tea trees. During the application of sheep manure fertilizer, a reasonable depth of ditching and burying was necessary to ensure soil health, which was conducive to the proliferation of the root system and the efficient absorption of nutrients by tea trees, thus improving the yield and quality of tea trees. It is particularly important to control the depth of fertilizer application during long-term use. Currently, there are few reports on research in this area.

Accordingly, in this study, tea plantations with severely acidified soils were selected as experimental sites, and sheep manure fertilizer was applied at different depths, respectively. The varieties of tea trees planted in tea plantations were all Tieguanyin. Tea yield and quality, physiological indexes of tea trees, pH, nitrogen conversion enzyme activity, microbial quantity, biochemical intensity and differential gene expression of soil were determined for 5 consecutive years (2018-2022). On this basis, the improvement effect of different depths of sheep manure fertilization on soil acidification in tea plantations, the effects on tea yield, tea quality and soil nitrogen transformation, and the interaction between different indexes were analyzed. The results of the study are intended to provide technical guidance for the improvement of acidified tea plantation soils and the regulation of tea plantation fertilization.

## 2 Materials and methods

### 2.1 Experimental tea plantation

**Figure 1:**
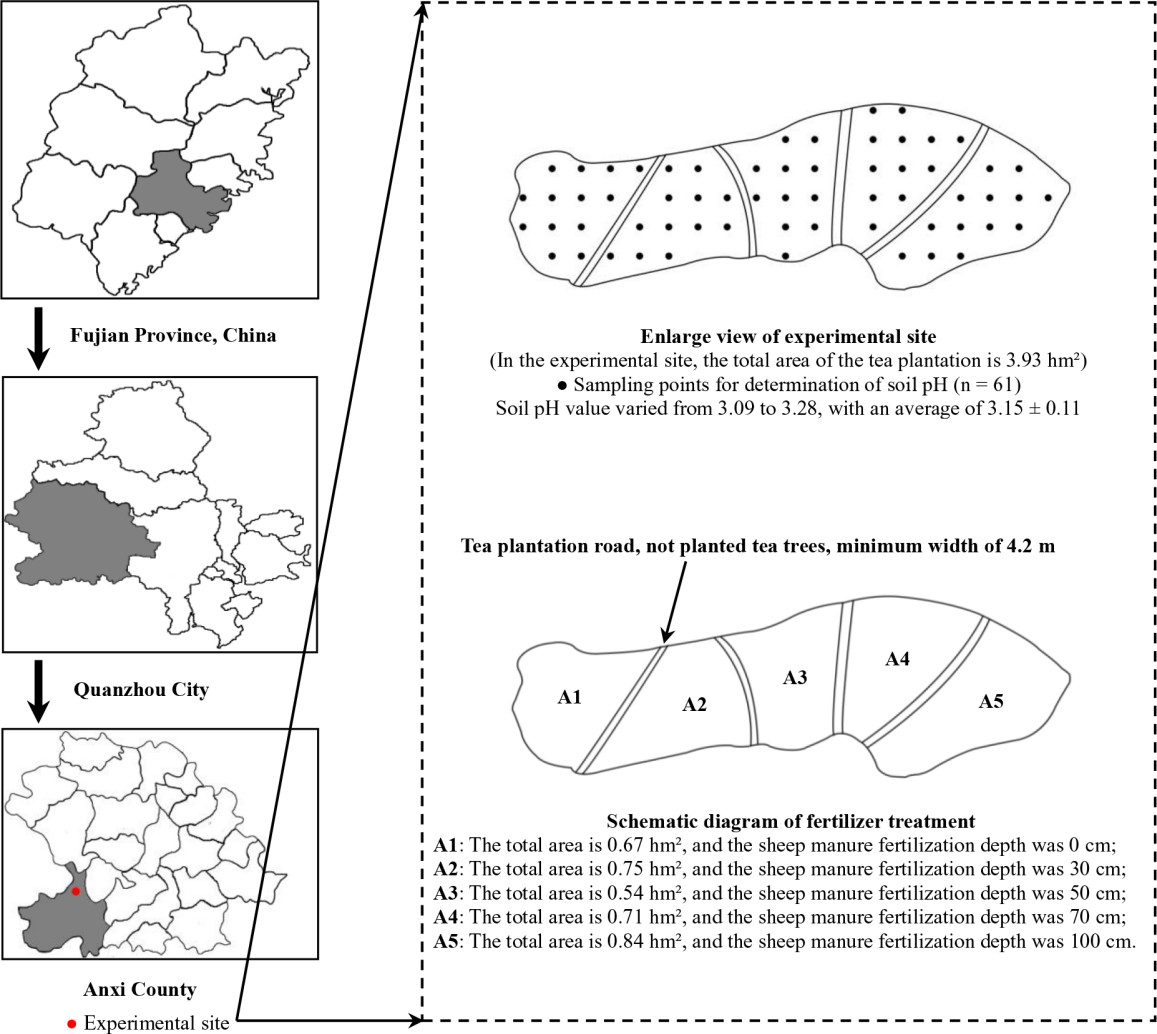
Schematic diagram of the experimental site and tea plantation zoning from 2017 to 2022.

Soils with pH less than 4.0 were severely acidified and unsuitable for tea tree cultivation (Mehra and Baker, 2017; Lin et al., 2022b). Based on our team’s previous studies (Wang et al., 2018; Wang et al., 2022c), the present study used a Tieguanyin tea plantation in Longjuan Township, Anxi County, Quanzhou City, Fujian Province (Longitude 117°93’ east and latitude 24°97’ north) as the experimental site. This experimental site was fixed for our team for a long time, and the tea plantation was centrally managed for fertilization, weeding, irrigation, disease and pest control. Among them, only sheep manure fertilizer was used, and the specific application method was carried out according to the experimental design. The tea plantation was manually weeded three times a year, in March, July and September respectively; the tea plantation was irrigated using an intelligent irrigation system, so that the water content of the tea plantation soil was maintained at about 30% on sunny days; the pest and disease control in the tea plantation was based on organic pesticides, the main organic pesticides used were matrine and azadirachtin. Each organic pesticide was used four times a year in March, July, August and September. Two organic pesticides were used alternately, and the use time interval was 10 days. The average altitude of the experimental site was 600 m, the average annual rainfall was 1,800 mm, the average annual relative humidity was about 80%, and the average annual temperature was 18 °C. The total area of the experimental tea plantation was 3.93 hm², the tea trees were 4-year-old Tieguanyin, and the average pH value of the tea plantation soil was 3.15, which was severely acidified, and other indexes were shown in **Table S1**. The experimental tea plantations were divided into five areas, namely A1, A2, A3, A4 and A5, in which the area of A1 tea plantation was 0.67 hm², A2 tea plantation was 0.75 hm², A3 tea plantation was 0.54 hm², A4 tea plantation area 0.71 hm², and A5 tea plantation area 0.84 hm² (**Figure 1**).

### 2.2 Fertilization treatment method and sample collection

The decomposed sheep manure was used as fertilizer (1.64% total nitrogen, 0.91% total phosphorus and 0.89% total potassium), and sheep manure fertilizer purchased from China Inner Mongolia Guwoheni Agricultural Technology Co., Ltd. The recommended dosage of sheep manure per hectare was 15 t/hm² in organic tea plantations according to Wang et al. (2020). The application depth of sheep manure fertilizer was 0 cm, 15-30 cm (30 cm), 15-50 cm (50 cm), 15-70 cm (70 cm), and 15-100 cm (100 cm) in A1, A2, A3, A4, and A5 tea plantations, respectively. The application method of sheep manure fertilizer was shown in **Figure S1**. During the experimental period (2017 to 2022), only sheep manure fertilizer was used in the tea plantation, and fertilizer was applied once a year between September 20 and 30, with the first application on September 26, 2017, and experimental indicators were measured in May of the following year (tea picking season).

In this study, tea yields from A1, A2, A3, A4, and A5 tea plantations were measured in May 2018, 2019, 2020, 2021, and 2022 after different fertilization treatments, while tea tree leaves (second leaves), root, and rhizosphere soil were collected from these tea plantations. The root and rhizosphere soil of tea trees were collected by removing fallen leaves from the surface layer of the soil and excavating the soil covering the upper layer up to the root of the tea tree layer by layer about 60 cm deep and excavating the whole tea tree, collecting soil around the root of the tea tree, which was rhizosphere soil, and collecting the root of the tea tree at the same time.

The tea tree leaves collected were used to determine amino acid and theanine content, the tea tree root was used to determine root activity and total root nitrogen content, and the tea rhizosphere soil was used to determine soil pH, ammonium nitrogen and nitrate nitrogen content, the number and intensity of ammonifying and nitrifying bacteria, soil enzyme activity, and soil gene expression.

### 2.3 Determination of physicochemical index

Soil pH was measured using a pH meter (PB-10, Sartorius), which was briefly described as: soil - water ratio of 1:2.5, shaking for 30 min, standing for 30 min, directed determination, 5 replicates per sample.

Root activity of tea trees was determined using the plant root activity detection kit (naphthalamine microplate method) provided by Beijing Leagene Biotechnology Co., Ltd. (Gui et al., 2022). Total root nitrogen content was determined by the Kjeldahl method with 5 replicates per sample.

The determination of tea yield was based on the method of Wang et al. (2020). Specifically, the measurement time was in May each year, the harvesting standard was 3-4 leaves in the middle and small open surface of the standing bud, the area of each sample was 10 m^2^ (1 row, Length 10 m × width 1 m), and all 10 m^2^ were planted with tea trees. Triplicate samples were determined for each fertilized tea plantation. The measured tea yield was converted into tea yield per hectare of tea plantation.

The contents of amino acids and theanine in fresh tea leaves were determined with 5 replicates for each sample. Amino acids content was determined by spectrophotometry and reaction with ninhydrin (General Administration of Quality Supervision, Inspection and Quarantine of the People’s Republic of China, 2014). Theanine content was determined according to the National Standard of the People’s Republic of China – GBT23193-2017, Determination of theanine in tea using high performance liquid chromatography (General Administration of Quality Supervision, Inspection and Quarantine of the People’s Republic of China, 2018).

The content of nitrate nitrogen and ammonium nitrogen in soil was determined by Li et al. (2021). Briefly, soil was extracted with 2 mol/L KCl solution for 1 h and filtered with a 0.45 μm membrane, and then soil extract was obtained and used to following measurement. The content of ammonium and nitrate in solution was determined by continuous flow analyzer (SA5000, Skalar Company, Netherlands) and converted into soil nitrogen content.

### 2.4 Determination of the number of soil nitrogen transforming bacteria

The number of soil nitrogen transforming bacteria was mainly determined by ammonifying bacteria and nitrifying bacteria according to Li et al. (2008). Among them, soil ammonia bacteria were cultured using liquid ammonia peptone medium (KH_2_PO_4_ 0.5 g, MgSO_4_-7H_2_O 0.5 g, peptone 5 g, distilled water 1 L, pH 7.0-7.2); ammonia bacteria were determined by MPN dilution method. Soil nitrifying bacteria were cultured using Stephenson’s modified medium with (NH_4_)_2_SO_4_ 2 g, K_2_HPO_4_ 0.75 g, NaH_2_PO_4_ 0.25 g, MgSO_4_-7H_2_O 0.03 g, CaCO_3_ 5 g, MnSO_4_-4H_2_O 0.01 g, agar 20 g, distilled water 1 L, pH 7.2; nitrifying bacteria were determined by plate colony counting method.

### 2.5 Determination of the intensity of soil biochemical processes

The intensity of soil biochemical processes was mainly determined by the intensity of ammonification and nitrification, and the determination method was referred to Li et al. (2008). Soil ammonia intensity was determined using medium culture of ammonium bacteria and soil suspension inoculation. Soil ammonia intensity was assessed by NH_4_-N content in 100 mL culture solution. Soil nitrification intensity using liquid nitrifying medium culture was determined by soil suspension inoculation method. The calculation method was as follows:


NI=(NCO−NCA)/NCO×100%

Where NI denotes nitrification intensity, NCO denotes nitrite content in the original medium and NCA denotes nitrite content in the medium after incubation.

### 2.6 Determination of soil enzyme activity

In this study, 4 enzymes involved in soil nitrogen transformation were determined by Enzyme Linked Immunosorbent Assay Kit, namely urease, protease, nitrate reductase, and nitrite reductase. Specific determination method: 1 g fresh soil was extracted with PBS buffer solution (soil - water ratio was 1:10); thereafter, ELISA enzyme-linked immunoassay kit (Beijing Huadeboyi Biological Technology Co., LTD.) was used to extract, and the absorbance (OD) value at 450 nm of the extraction solution was detected by a multifunctional enzyme marker (BioTek Synergy2, US). The results of enzyme activity were expressed as the molar mass (μmol) of the enzyme produced per unit volume (L^-1^) and per unit time (min^-1^). The kit principle applied a double antibody clamping method to determine the level of enzyme activity in soil samples. As an example of nitrite reductase determination, the purified nitrite reductase antibody was coated with microporous plates to produce solid phase antibodies; the extracted test solution was added to the micropores coated with monoclonal antibody. In addition, horseradish peroxidase (HRP)-labeled nitrite reductase antibody was added to form the antibody-antigen-enzyme-labeled antibody complex. Thereafter, the compound was thoroughly washed and tetramethyl benzidine (TMB) was added for color development. TMB was converted to blue under the catalysis of HRP enzyme, and finally to yellow under the action of acid. Color depth was positively correlated with nitrite reductase in the sample. Other enzymes were determined using a similar method.

### 2.7 Gene expression analysis

In this study, 6 genes related to soil nitrogen transformation were determined by quantitative fluorescence PCR, namely *nifH*, *amoA*-AOA, *nirK*, *nirS*, *narG*, and *nosZ* genes.

PowerSoil™ Total DNA Isolation Kit was used to extract total DNA from soil microorganisms. DNA was detected by 1% agarose electrophoresis, and DNA concentration and purity were determined by microspectrophotometer, after then, the extracted DNA was stored at -20°C.

The primer design of genes related to soil nitrogen transformation was shown in **Table 1** (Liao et al., 2019). The PCR system was 50 μL, including 2×*Fine Taq*™ PCR supermix 25 μL, DNA template 1 μL, forward primer 1 μL (10 μmol/L), reverse primer 1 μL (10 μmol/L), and 22 μL of ddH_2_O. The amplification procedure was 98°C for 10 s, 52°C for 30 s, 72°C for 30 s, 35 cycles, finally, it was kept at 72°C for 5 min (Ding et al., 2022). During the operation of this experiment, each sample had 3 replicates, and the gene copy number was determined and converted by ABI 7500 real-time quantitative fluorescence PCR system. At the same time, the corresponding plasmids were set with concentration gradients of 0.001, 0.005, 0.01, 0.05, 0.1, 0.5, 1, and 2 ng. After amplification with a quantitative fluorescence PCR instrument (ABI 7500, USA), standard curves were drawn with Ct value as abscate and log10 (copy number) as ordinate. The measured Ct values of the samples were compared with standard curves to calculate microbial gene expression per gram of dry soil.

**Table 1:** Primers of genes related to soil nitrogen transformation.

Gene	Forward primer	Reverse primer	Function
nif*H*	AAAGGYGGWATCGGYAARTCCACCAC	TTGTTSGCSGCRTACATSGCCATCAT	N_2_→NH_3_
amo*A*-AOA	CCCCTCKGSAAAGCCTTCTTC	GCCATCCATCTGTATGTCCA	NH_3_→NH_2_OH
nar*G*	TAYGTSGGGCAGGARAAACTG	CGTAGAAGAAGCTGGTGCTGTT	$NO_{3}^{\;}-\to NO_{2}^{\;}-$
nir*K*	ATYGGCGGVCAYGGCGA	GCCTCGATCAGRTTRTGGTT	$NO_{2}^{\;}-\to NO^{-}$
nir*S*	GTSAACGYSAAGGARACSGG	GASTTCGGRTGSGTCTTSAYGAA	$NO_{2}^{\;}-\to NO^{-}$
nos*Z*	AGAACGACCAGCTGATCGACA	TCCATGGTGACGCCGTGGTTG	N_2_O^−^→N_2_

### 2.8 Statistical analysis

Excel 2017 software was used to calculate the mean value and variance of the data, Origin 2018 software was used to make radar maps, trend charts, violin charts and box charts, Rstudio 3.3 software was used to make principal component maps, Cytoscape_v3.9.1 software was used to make correlation analysis charts. The TOPSIS entropy weight statistical analysis was performed on the SPSSAU online platform (https://spssau.com/).

## 3 Results and discussion

### 3.1 Effects of sheep manure fertilizer with different application depths on soil pH and tea yield and quality

**Figure 2:**
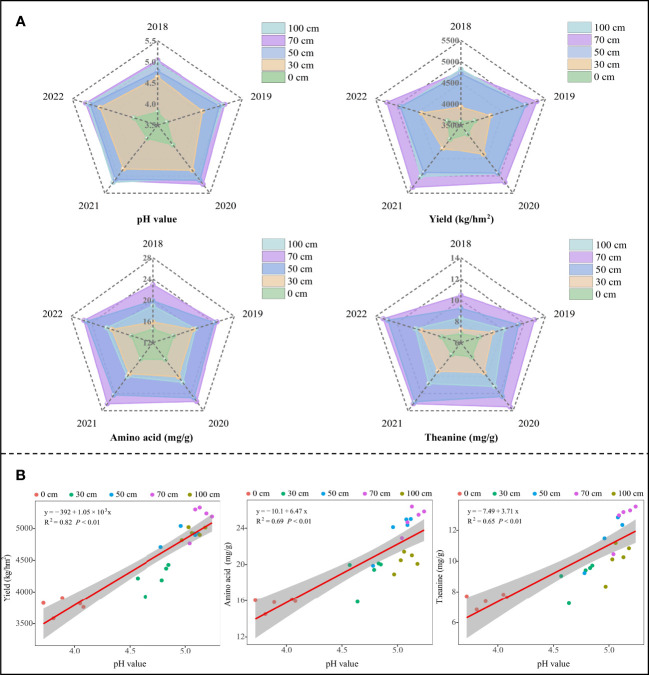
Effect of sheep manure fertilizer on soil pH value, tea yield and quality. **(A)** Effect of sheep manure fertilizer with different depths on soil pH value, tea yield and quality from 2018 to 2022; **(B)** Effect of soil pH on tea yield and quality from 2018 to 2022.

In this study, we analyzed the effects of sheep manure fertilizer at different depths on soil pH and tea yield and quality from 2018 to 2022, and the results showed (**Figure 2A**) that sheep manure fertilizer could effectively increase soil pH, tea yield, and amino acid and theanine content in tea leaves. Secondly, as the application depth of sheep manure increased (from 0 cm to 100 cm), soil pH, tea yield, and amino acid and theanine content in tea leaves showed a trend of increasing and then decreasing, all reaching maximum values at the application depth of 70 cm. Sheep manure fertilizer plays an important role in agricultural production as an organic fertilizer. Many scholars have studied the effects of sheep manure fertilizer on soil improvement and crop growth, and found that sheep manure fertilizer significantly reduced soil acidification (*P*< 0.05), increase soil pH, and promote crop growth (Souza et al., 2021; Traoré et al., 2021). The present study also found that sheep manure fertilizer could alleviate soil acidification and improve tea yield and quality in tea plantations, but there were differences in the effects of the depth of application of sheep manure fertilizer on tea tree growth. After sheep manure fertilizer was ditched and buried, the site where it was buried had rapid soil improvement and the best nutrient supply, while other areas could only gradually improve by relying on the long-term decomposition and migration of sheep manure fertilizer. Therefore, the root length of different crops determines the depth of fertilizer application. The root was the main part of nutrient uptake by the tea tree, and the root of adult oolong tea trees was about 50-80 cm, and the depth of fertilizer application was extremely important for whether the root of the tea tree could fully absorb nutrients (Hu et al., 2020; Shao et al., 2021). In this study, it was found that the fertilizer application depth of 70 cm was the most favorable for the growth of the tea tree and the yield and quality of the tea leaves were the highest. It can be seen that effective adjustment of fertilizer application depth could promote the growth of tea trees.

After continuous application of sheep manure fertilizer, the relationship between soil pH and tea yield and quality was further analyzed, and the results showed (**Figure 2B**) that soil pH was significantly and positively correlated with tea yield (R^2 =^ 0.82, *P*< 0.01), amino acid content (R^2^ = 0.69, *P*< 0.01) and theanine content (R^2^ = 0.65, *P*< 0.01) of tea leaves, and tea yield, amino acid and theanine content in tea leaves showed a significant upward trend with the increase in pH value. The soil pH suitable for tea tree growth was from 4.5 to 5.5, and when the soil pH was below 4.5, tea tree growth was hindered and tea leaf yield and quality decreased (Wang et al., 2018; Wang et al., 2022c). It can be seen that sheep manure fertilization can effectively alleviate soil acidification in tea plantations and increase soil pH, which in turn promotes the growth of tea trees and improves tea yield and quality.

### 3.2 Effect of sheep manure fertilizer with different application depths on root activity and total root nitrogen content of tea trees

**Figure 3:**
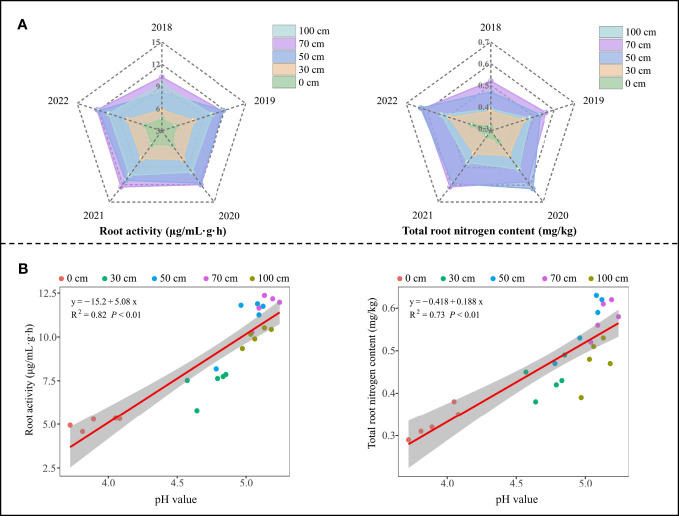
Effect of sheep manure fertilizer and soil pH on root activity and root total nitrogen content of tea tree. **(A)** Effect of sheep manure fertilizer with different depths on root activity and total root nitrogen content of tea trees from 2018 to 2022; **(B)** Effect of soil pH on root activity and total root nitrogen content of tea trees from 2018 to 2022.

Root was the main part of plant nutrient uptake, and root activity directly affected plant nutrient uptake capacity, and high root activity was beneficial to plant nutrient uptake and accumulation, which was beneficial to plant growth, and vice versa (Moretti et al., 2020; Chen et al., 2022). In this study, it was found (**Figure 3A**) that sheep manure fertilizer was beneficial to root activity and total root nitrogen content of tea trees, and the two indexes generally showed an increasing trend with the extension of sheep manure fertilization time (2018-2022). Secondly, the analysis revealed that the root activity and total root nitrogen content of tea trees remained at a high level when the application depth of sheep manure fertilizer was 50 cm and 70 cm (**Figure 3A**). The method of using sheep manure fertilizer in tea plantations was to bury it in trenches around the root system of tea trees, so that the root system of tea trees could be exposed to sheep manure fertilizer faster and in a larger area, which was conducive to promoting the growth of tea tree roots, improving root activity, and thus improving the nutrient absorption ability of tea trees, while the root system of adult oolong tea trees ranged from 50-80 cm (Hu et al., 2020; Shao et al., 2021). It can be seen that when sheep manure was applied at a depth of the root growth range, tea trees could directly and effectively obtain nutrients, which in turn promoted root growth and improved root activity.

Furthermore, the growth and activity of tea tree root systems were closely related to pH value. Sun et al. (2020) adopted the hydroponics method to adjust the pH of the culture medium and analyze the effect of pH on tea tree growth. The results showed that when the pH of the culture medium was greater than 4.5, the root growth of tea trees was favored and the number and area of new roots increased significantly, which in turn improved the uptake and utilization of nutrients by tea trees. Secondly, amino acids and theanine as indexes of tea quality were nitrogenous compounds, and their synthesis in plants was closely related to the nitrogen uptake capacity of tea trees (Li et al., 2019; Wang et al., 2021). The increase in root activity of tea trees may be conducive to the uptake and accumulation of more nitrogen by tea trees, which in turn laid the foundation for the synthesis of more amino acids and theanine. This study found (**Figure 3B**) that soil pH was significantly and positively correlated with root activity (R^2^ = 0.82, *P*< 0.01) and total root nitrogen content (R^2^ = 0.73, *P*< 0.01) of tea trees from 2018 to 2022 after continuous use of sheep manure fertilizer, i.e., root activity and total root nitrogen content of tea trees showed a significant increasing trend with the increase of soil pH. Sheep manure fertilizer is rich in organic matter, which could reduce the loss of alkaline substances from the soil and improve soil buffering capacity against acidification (Le et al., 2022). The contact between plant roots and sheep manure fertilizer could accelerate the decomposition of soil organic matter, improve the efficiency of nutrients decomposition and release, and increase the pH value of soil (Yao et al., 2009). As mentioned above, the root system of adult tea trees ranged from 50 to 80 cm. The depth of application of sheep manure fertilizer determined the range of soil that it could directly improve, and the closer the depth of application was to the root system of the tea tree, the faster the soil pH of the root would increase, and the more beneficial it would be to root growth and nutrient absorption. It can be seen that the use of sheep manure fertilizer was conducive to alleviating soil acidification in tea plantations, raising soil pH, promoting root development and improving nitrogen uptake and utilization capacity of tea trees, thereby improving tea yield and quality, especially when the fertilization depth was 50-70cm.

### 3.3 Effect of sheep manure fertilizer with application depth on soil nitrogen transforming microorganisms

**Figure 4:**
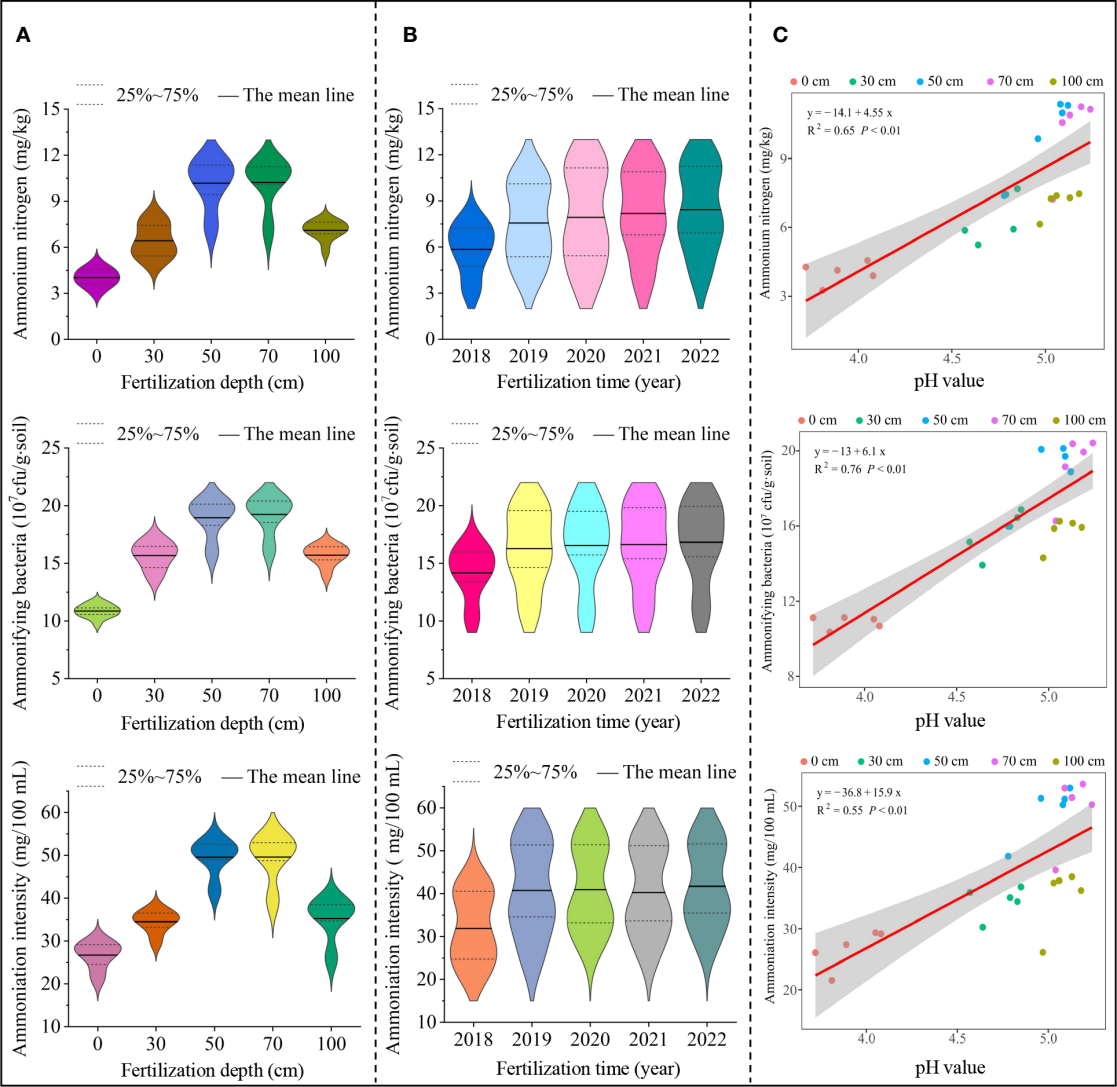
Effect of different application depths of sheep manure fertilizer and soil pH on soil ammonium transformation. **(A)** Effect of different application depths of sheep manure fertilizer on ammonium nitrogen content, ammonifying bacteria number, and ammoniation intensity; **(B)** Effect of different application depths of sheep manure fertilizer on ammonium nitrogen content, ammonifying bacteria number, and ammoniation intensity from 2018 to 2022; **(C)** Effect of soil pH on ammonium nitrogen content, ammonifying bacteria number, and ammoniation intensity from 2018 to 2022.

**Figure 5:**
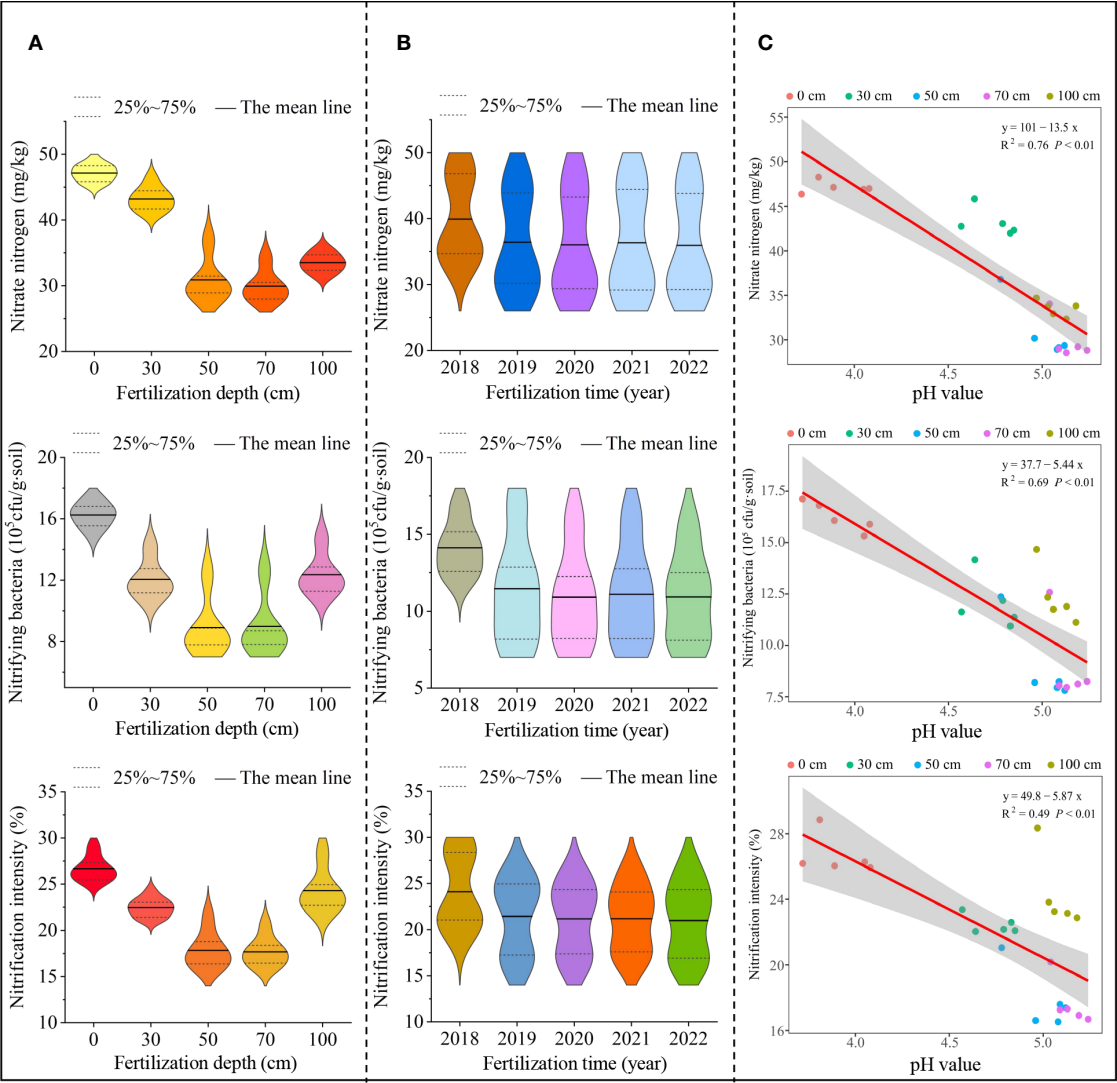
Effect of different application depths of sheep manure fertilizer and soil pH on soil nitrate transformation. **(A)** Effect of different application depths of sheep manure fertilizer on nitrate nitrogen content, nitrifying bacteria number, and nitrification intensity; **(B)** Effect of different application depths of sheep manure fertilizer on nitrate nitrogen content, nitrifying bacteria number, and nitrification intensity from 2018 to 2022; **(C)** Effect of soil pH on nitrate nitrogen content, nitrifying bacteria number, and nitrification intensity from 2018 to 2022.

Tea trees were mainly harvested from young shoots and leaves; therefore, tea tree growth had a high demand for fertilizers, especially nitrogen (Liu et al., 2018). Soil nitrogen content and nitrogen transformation capacity directly affected the yield and quality of tea leaves (Li et al., 2020b). Plants mainly take up soil nitrogen in the form of inorganic nitrogen, containing ammonium and nitrate nitrogen (Zhang et al., 2019a). The transformation of different forms of nitrogen in soil and their contents were closely related to the number and intensity of soil microorganisms, e.g., ammonifying bacteria could convert organic and mineralized nitrogen in soil into ammonium nitrogen, while nitrifying bacteria could convert ammonium nitrogen in soil into nitrate nitrogen (Yan et al., 2020a; Yu et al., 2022). Tea trees were ammonium-loving plants, and ammonium nitrogen was beneficial to root development and increased the accumulation of free amino acids in tea leaves, which was beneficial to the improvement of tea growth and quality, while high nitrate nitrogen content would inhibit tea tree growth (Duan et al., 2019; Li et al., 2020b). In this study, it was found that with the increase of the application depth of sheep manure fertilizer (0-100 cm), the soil ammonium nitrogen content, the number of ammonifying bacteria and the ammonification intensity showed a trend of increasing and then decreasing, with the maximum at 50 cm and 70 cm (**Figure 4A**). Secondly, soil ammonium nitrogen content, ammonification bacteria number and ammonification intensity showed an overall increasing trend with the extension of fertilizer application time (2018 to 2022) (**Figure 4B**). Further analysis revealed that with the increase of the depth of application of sheep manure fertilizer (0 - 100 cm), soil nitrate nitrogen content, nitrifying bacteria population and nitrification intensity showed a decreasing and then increasing trend, with the minimum at 50 cm and 70 cm (**Figure 5A**). Secondly, soil nitrate nitrogen content, nitrifying bacteria number and nitrification intensity showed a decreasing trend overall with the extension of fertilizer application time (2018 to 2022) (**Figure 5B**). It can be seen that the use of sheep manure fertilizer reduced the transformation capacity of soil nitrate nitrogen and favored the transformation and accumulation of ammonium nitrogen, thus promoting the growth of tea tree roots. Better results could be achieved by reasonably controlling the application depth of sheep manure fertilizer.

Changes in pH were likely to affect soil nitrogen transformation. For example, in soils with lower pH, nitrifying bacteria were more likely to survive and multiply, while ammonifying bacteria did the opposite, leading to an increase in soil nitrate nitrogen and a decrease in ammonium nitrogen (Li et al., 2020a; Takahashi et al., 2020; Scarlett et al., 2021). In this study, we found (**Figure 4C**) that soil pH was significantly and positively correlated with soil ammonium nitrogen content (R^2^ = 0.65, *P*< 0.01), ammonifying bacteria number (R^2^ = 0.76, *P*< 0.01) and ammonification intensity (R^2^ = 0.55, *P*< 0.01) after continuous use of sheep manure fertilizer from 2018 to 2022, while it was significantly and positively correlated with soil nitrate nitrogen content (R^2^ = 0.76, *P*< 0.01), nitrifying bacteria number (R^2^ = 0.69, *P*< 0.01) and nitrification intensity (R^2^ = 0.49, *P*< 0.01) (**Figure 5C**). It can be seen that the long-term use of sheep manure fertilizer was beneficial to alleviate soil acidification, improve soil pH, increase the number and intensity of soil ammoniating bacteria, and promote the transformation and accumulation of soil ammonium nitrogen in tea plantations, so as to be absorbed and used by tea trees.

### 3.4 Effect of sheep manure fertilizer with different depths on enzyme activity related to soil nitrogen transformation

**Figure 6:**
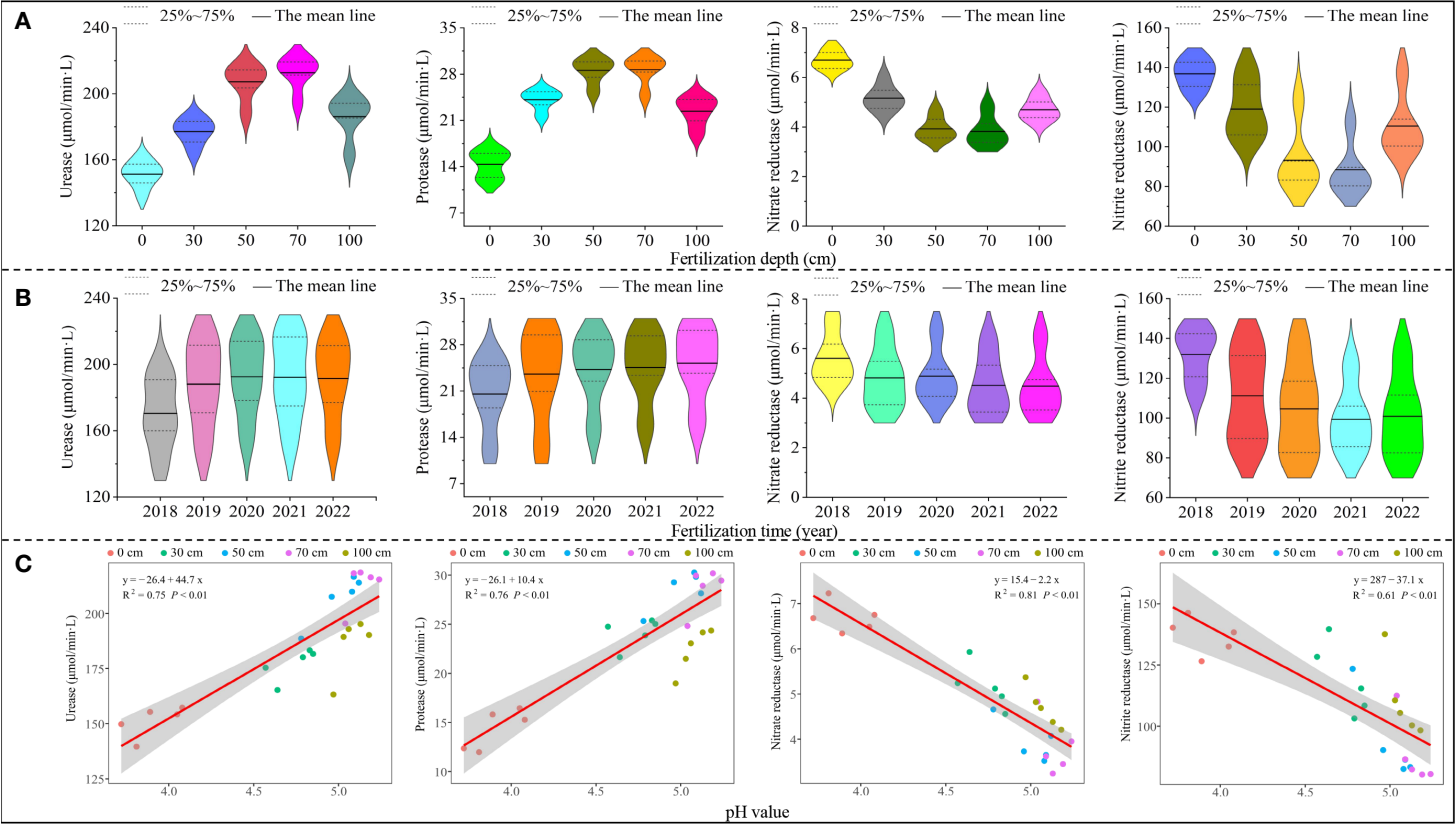
Effect of different application depths of sheep manure fertilizer and soil pH on soil enzyme activity. **(A)** Effect of different application depths of sheep manure fertilizer on the activity of urease, protease, nitrate reductase, and nitrite reductase; **(B)** Effect of different application depths of sheep manure fertilizer on the activity of urease, protease, nitrate reductase, and nitrite reductase from 2018 to 2022; **(C)** Effect of soil pH on the activity of urease, protease, nitrate reductase, and nitrite reductase from 2018 to 2022.

Soil nitrogen transformation was closely related to soil enzyme activity, which in turn reflected the direction and intensity of soil biochemical processes and nutrient cycling. For example, soil urease promoted the transformation of soil organic nitrogen to ammonium nitrogen (Zhao et al., 2022), protease hydrolyzed organic nitrogen to amino acids (Greenfield et al., 2021), and nitrate reductase and nitrite reductase converted soil nitrogen to nitrate nitrogen (Koyama et al., 2020). In this study, it was found that with the increase in application depth of sheep manure fertilizer (0-100 cm), soil urease and protease activities showed a trend of increasing and then decreasing, while nitrate reductase and nitrite reductase showed the opposite trend, with the greatest effect on soil enzyme activities at application depths of 50 cm and 70 cm (**Figure 6A**). Secondly, it was found that soil urease and protease activities showed an overall increasing trend, while nitrate reductase and nitrite reductase showed a decreasing trend with longer application time of sheep manure fertilizer (from 2018 to 2022) (**Figure 6B**). It can be seen that the use of sheep manure fertilizer was conducive to the improvement of enzyme activities related to soil ammonium nitrogen conversion, and thus increased soil ammonium nitrogen content, which also verified the above results.

Further analysis revealed that after long-term use of sheep manure fertilizer (2018 to 2022), soil pH was significantly and positively correlated with activities of soil urease (R^2^ = 0.75, *P*< 0.01) and protease (R^2^ = 0.76, *P*< 0.01), while it was significantly and positively correlated with activities of nitrate reductase (R^2^ = 0.81, *P*< 0.01) and nitrite reductase (R^2^ = 0.61, *P*< 0.01) (**Figure 6C**). It was reported that low pH was highly likely to enhance soil nitrate reductase and nitrite reductase activities and inhibit soil urease and protease activities, which in turn increased soil nitrification capacity, decreased ammonification capacity, increased soil nitrate nitrogen content, and decreased ammonium nitrogen content (Ding et al., 2022; Lin et al., 2022a). It can be seen that the effect of sheep manure fertilizer on soil nitrogen conversion lay in the adjustment of soil pH, increasing soil pH value was conducive to the transformation of soil ammonium nitrogen and vice versa for the transformation of nitrate nitrogen.

### 3.5 Effects of sheep manure fertilizer with different application depths on the expression of genes related to soil nitrogen transformation

**Figure 7:**
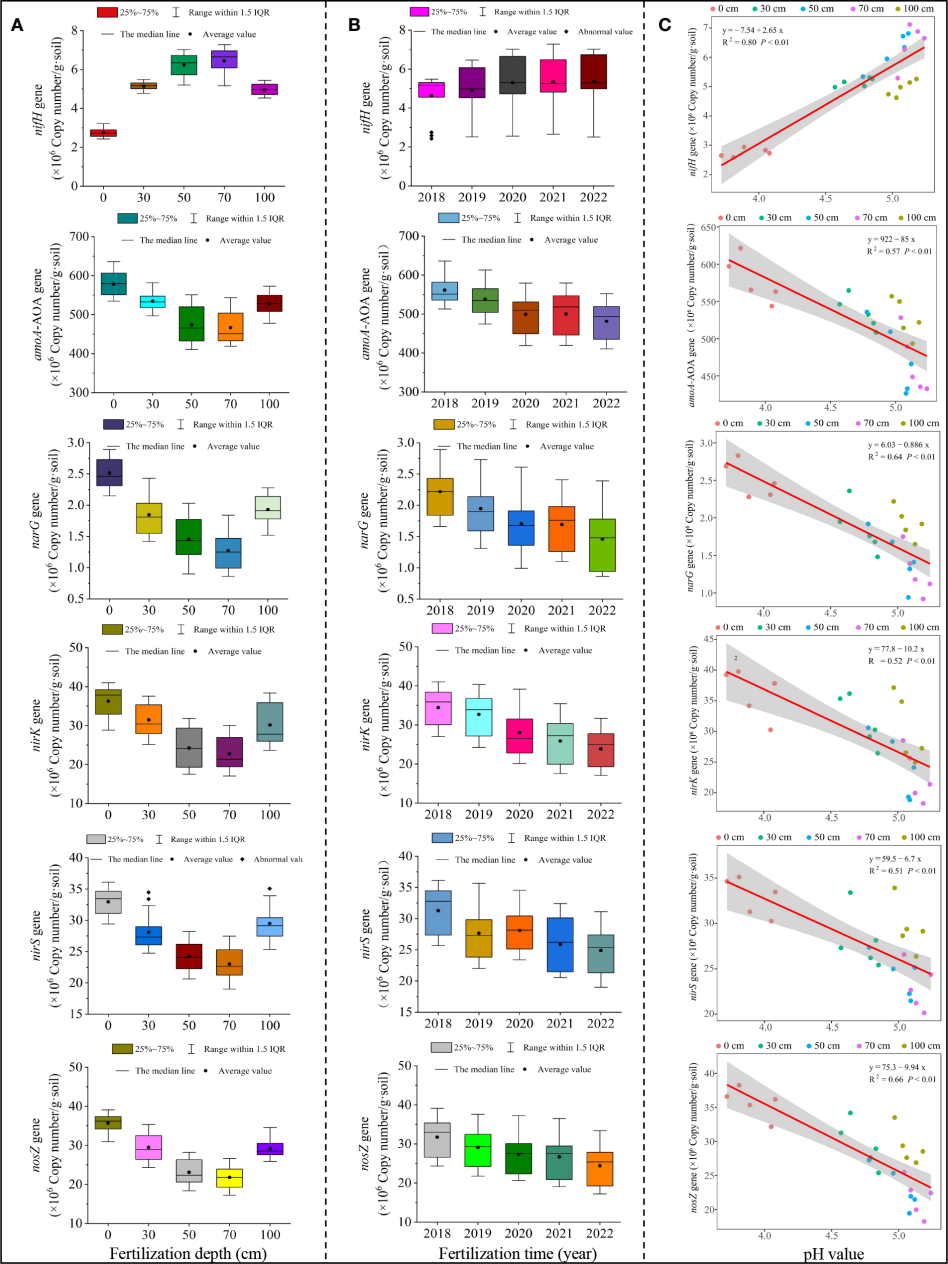
Effect of different application depths of sheep manure fertilizer and soil pH on the expression of genes related to soil nitrogen transformation. **(A)** Effect of different application depths of sheep manure fertilizer on the expression of genes related to soil nitrogen transformation; **(B)** Effects of different application depths of sheep manure fertilizer on the expression of genes related to soil nitrogen transformation from 2018 to 2022; **(C)** Effect of soil pH on the on the expression of genes related to soil nitrogen transformation from 2018 to 2022.

Soil nitrogen transformation was performed by microbial populations containing specific functional genes. For example, microorganisms containing the *nifH* gene were able to convert airborne N_2_ to NH_3_, thus increasing the expression of the *nifH* gene facilitated soil ammonia nitrogen accumulation (Ospina et al., 2020). Microorganisms containing *amoA-AOA, nirK, nirS, narG*, and *nosZ* genes could oxidize NH^4+^ to NO^3-^ and then reduce NO^3-^ to NO^2-^, NO^-^, and even N_2_ (Maul et al., 2019; Zhang et al., 2019b; Rahman et al., 2020). Therefore, changes in the expression of these genes were often used to characterize ammonia and nitrification processes in soils. In this study, we found that the expression of soil *nifH* genes showed a trend of increasing and then decreasing with the increasing depth of application of sheep manure fertilizer (0-100 cm), while the expression of *amoA-AOA, narG, nirK, nirS* and *nosZ* genes showed a trend of decreasing and then increasing, and the peak expression of both types of genes reached about 50 cm and 70 cm of fertilizer application depth (**Figure 7A**). Further analysis revealed that the expression of *nifH* genes showed an overall increasing trend with the prolongation of sheep manure use (2018-2022), while the expression of *amoA-AOA, narG, nirK, nirS* and *nosZ* genes showed the opposite trend (**Figure 7B**). From the perspective of soil nitrogen conversion gene expression, the results found that the use of sheep manure fertilizer was beneficial to reduce soil nitrate nitrogen conversion capacity, improve soil ammonium nitrogen conversion capacity, and thus increase soil ammonium nitrogen content.

Further analysis of the effect of soil pH on the expression of genes related to soil nitrogen transformation showed (**Figure 7C**) that soil pH was significantly and positively correlated with the expression of soil *nifH* genes (R^2^ = 0.80, *P*< 0.01), while it was significantly and negatively correlated with the expression of soil *amoA*-AOA(R^2^ = 0.57, *P*< 0.01), *narG*(R^2^ = 0.64, *P*< 0.01), *nirK*(R^2^ = 0.52, *P*< 0.01), *nirS*(R^2^ = 0.51, *P*< 0.01)and *nosZ*(R^2^ = 0.66, *P*< 0.01)gene expression. It has been reported that the increase of soil pH was beneficial to enhancing *nifH* gene expression, enhancing soil ammonification capacity, and improving soil ammonium nitrogen accumulation (Ding et al., 2022; Dong et al., 2022). In contrast, soil pH increases decreased the expression of *amoA-AOA, narG, nirK, nirS* and *nosZ genes*, decreased the intensity of soil nitrification and denitrification, and thus decreased soil nitrate nitrogen content (Wang et al., 2022a; Yao et al., 2022). The results suggested that the key to the effect of sheep manure fertilizer on soil nitrogen conversion was soil pH regulation.

### 3.6 Principal component analysis and correlation analysis based on different indexes

**Figure 8:**
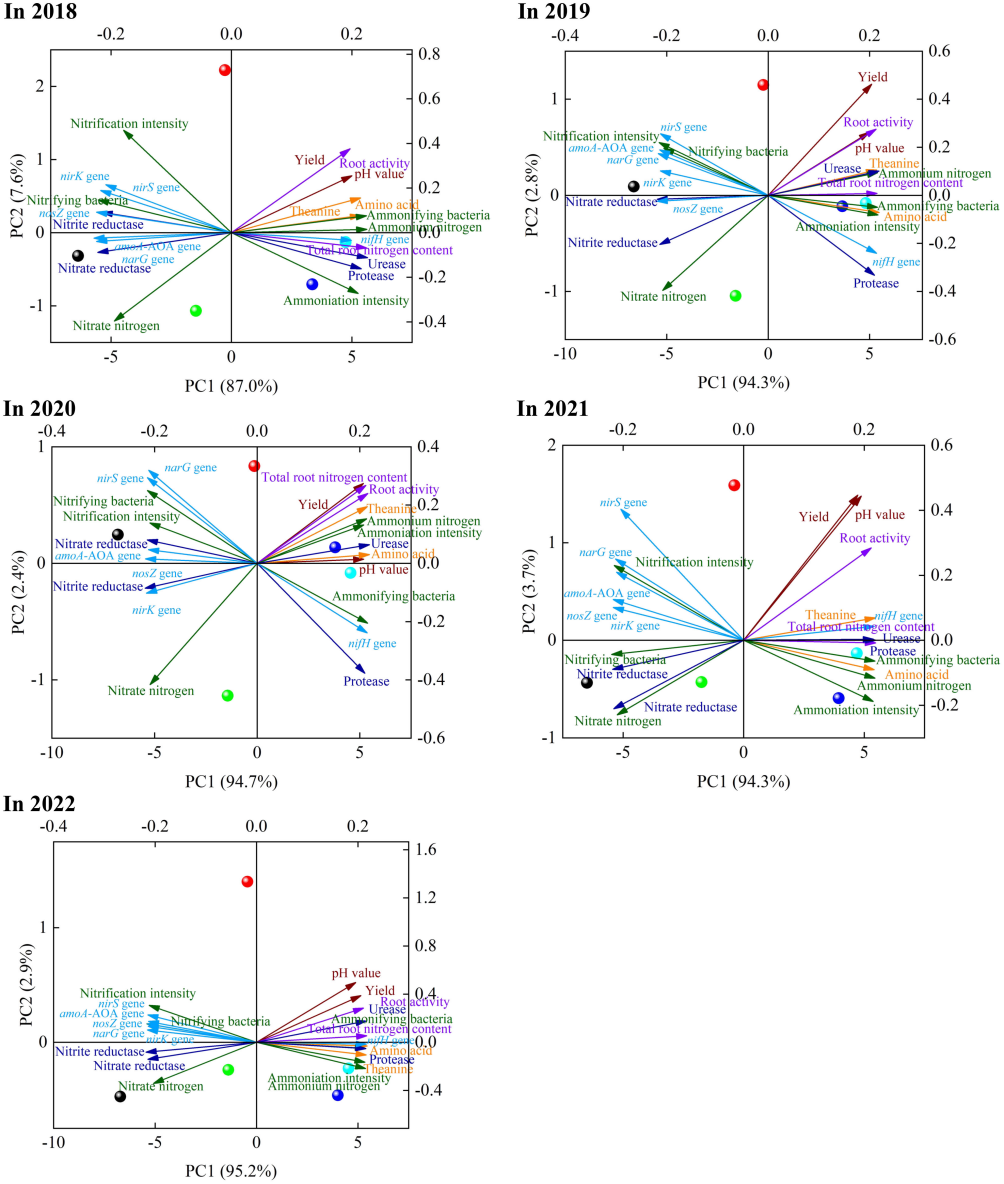
Principal component analysis of different indexes under sheep manure fertilizer with different depths. ●: Sheep manure was applied to a depth of 0 cm; ●: Sheep manure was applied to a depth of 30 cm; ●: Sheep manure was applied to a depth of 50 cm; ●: Sheep manure was applied to a depth of 70 cm; ●: Sheep manure was applied to a depth of 100 cm.

**Figure 9:**
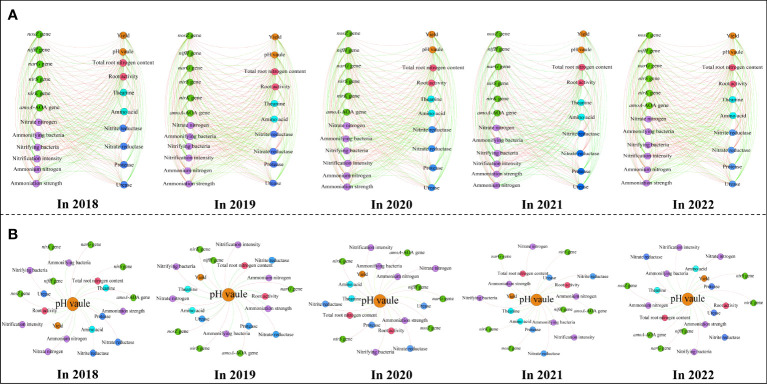
Interaction network between soil and tea indexes treated by sheep manure fertilizer with different depths based on correlation analysis. 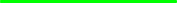 Positive correlation; 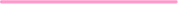
Negative correlation; **(A)** Interaction network between different indexes from 2018 to 2022; **(B)** Interaction network between soil pH and different indiexes from 2018 to 2022.

Based on the above analysis, this study further discussed the relationship between soil nitrogen conversion related indexes and tea yield and quality after application of sheep manure fertilizer. The results of principal component analysis based on different indexes showed (**Figure 8**) that different indexes formed two principal components after different depths of fertilization treatments of sheep manure fertilizer from 2018 to 2022, and the contribution of principal component 1 was the largest, which varied from 87.0% to 95.2% and could effectively distinguish different treatments. Second, treatments with fertilizer application depths of 0 cm, 30 cm and 100 cm were found at the negative end of principal component 1, while treatments with fertilizer application depths of 50 cm and 70 cm were found at the positive end of principal component 1 from 2018 to 2022. Further analysis revealed that the indexes concentrated at the negative end of principal component 1 were mainly nitrate nitrogen, nitrifying bacteria, nitrification intensity, nitrate reductase, nitrite reductase, *amoA*-AOA gene, *narG* gene, *nirK* gene, *nosZ* gene and *nirS* gene, which were mainly associated with soil nitrate nitrogen transformation (**Figure 8**), while the correlation analysis results showed (**Figure 9A**) a significant positive correlation between these indicators. The indexes concentrated at the positive end of principal component 1 were mainly pH value, tea yield, amino acid, theanine, root activity, total root nitrogen content, ammonium nitrogen, ammonifying bacteria ammoniation intensity, urease, protease, *nifH* gene, etc., and these indexers were mainly divided into three categories, including soil pH value, tea yield and quality, and soil ammonium nitrogen transformation and uptake capacity (**Figure 8**), whereas correlation analysis results showed (**Figure 9A**) that there was a significant positive correlation between these indexes. In addition, there was a significant negative correlation between indexes concentrated on the negative end of principal component 1 and indexes concentrated on the positive end of principal component 1 (**Figure 9A**). For example, soil pH was significantly negatively correlated with indexes concentrated on the negative end of principal component 1, while it was significantly positively correlated with indexes concentrated on the positive end of principal component 1 (**Figure 9B**). It can be seen that the key to distinguishing the effects of different fertilization depths of sheep manure fertilizer on tea yield and quality lay mainly in the influence of soil pH on the conversion capacity of soil ammonium nitrogen and nitrate nitrogen. Fertilization depths of 50 cm and 70 cm of sheep manure fertilizer were most conducive to alleviating soil acidification and conversion and accumulation of soil ammonium nitrogen, as well as improving root activity and root nitrogen absorption of tea trees, and most conducive to improving the yield and quality of tea trees.

**Figure 10:**
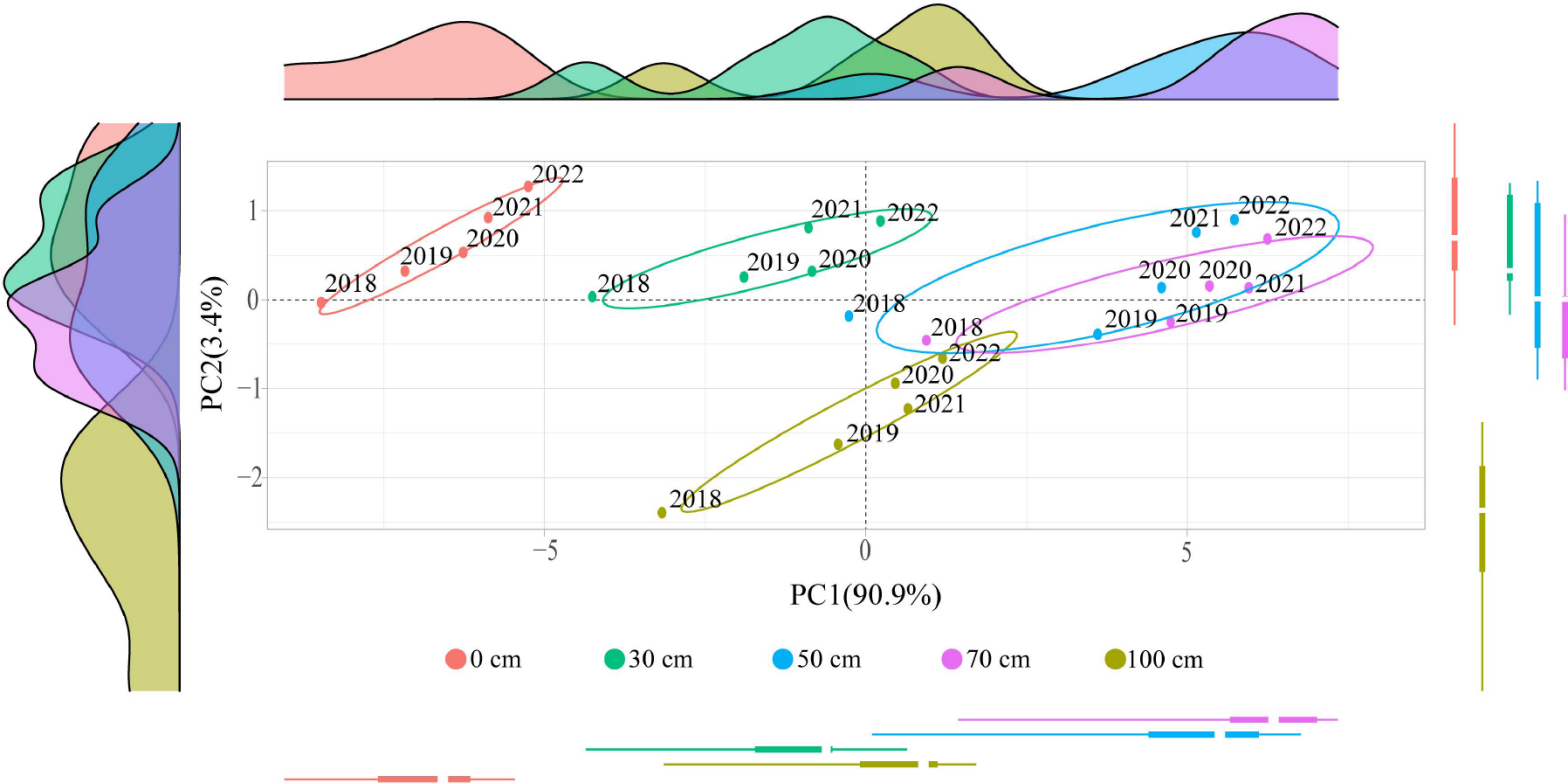
Principal component analysis of different years treated by sheep manure fertilizer with different depths.

The principal component analysis of the application time and depth of sheep manure fertilizer based on different indicators showed (**Figure 10**) that the contribution of principal component 1 reached 90.9%, which could effectively distinguish the treatments with different application depths of sheep manure fertilizer. The treatment with different application depths of sheep manure fertilizer was the worst when the application depth was 0 cm, followed by 30 cm and 100 cm, while the best results were obtained when the application depth was 50 cm and 70 cm. Tea trees are ammonium-loving plants, and the root was the main part of the nutrient absorption of tea trees, and the root system of adult oolong tea trees was about 50-80 cm, so the depth of fertilizer application was extremely critical to whether the root of tea trees could fully absorb nutrients (Hu et al., 2020; Shao et al., 2021). It can be seen that the use of sheep manure fertilizer was beneficial to improve root activity and increase the ammonium nitrogen content in the soil, which in turn promoted the uptake and accumulation of nitrogen by tea trees and improved tea yield and quality. However, it was necessary to control the application depth of sheep manure fertilizer during the fertilization process to achieve the best results.

### 3.7 Analysis of the effect of different indexes on fertilization effect under different application depths

**Figure 11:**
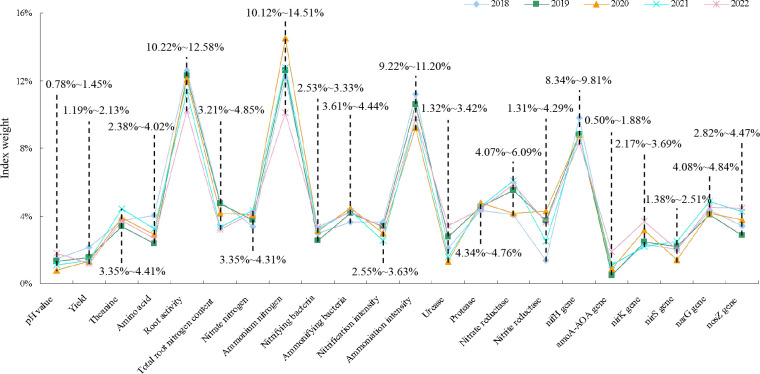
Topsis method analysis of the weight of effect of different indexes on fertilization.

The topsis method was a common and effective method in multi-objective decision analysis, which could be used to analyze the weight of different indexes on the effect of the results, where the greater the weight of the indexes, the stronger the influence of the indexes on the results, and vice versa (Al-Thani et al., 2022). This study used the topsis model to analyze the effect of different indexes on fertilization effect, and the results showed (**Figure 11**) that from 2018 to 2022, there were four main indexes that had strong effects on fertilization effect, namely root activity (10.22% to 12.58%), ammonium nitrogen (10.12% to 14.51%), ammoniation intensity (9.22% to 11.20%), and *nifH* gene (8.34% to 9.81%). It can be seen that the key to improving the fertilization effect of sheep manure fertilizer was whether it could improve the root activity of tea trees, improve the transformation capacity and accumulation of ammonium nitrogen in soil, and then improve the absorption capacity of tea trees for ammonium nitrogen, and finally improve the yield and quality of tea leaves.

## 4 Conclusion

**Figure 12:**
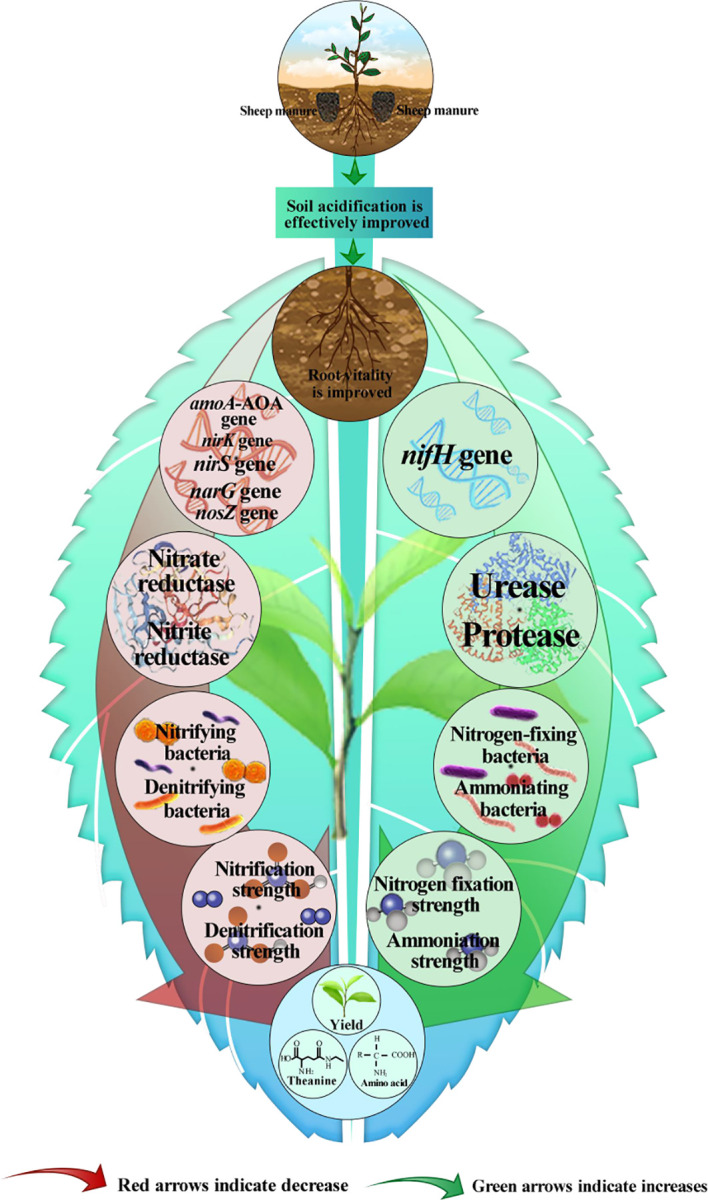
Mechanism diagram of effects of sheep manure fertilizer on soil nitrogen transformation.

This study analyzed the effect of sheep manure fertilizer with different application depths (0-100 cm) at the same dose on tea plantations with already acidified soils from 2018 to 2022, including soil pH, tea yield, quality, and soil nitrogen transformation. The results found (**Figure 12**) that the long-term use of sheep manure fertilizer was beneficial in alleviating soil acidification in tea plantations, improving soil pH and ammonium nitrogen content, enhancing root activity, which in turn led to an increase in the ability of tea tree roots to absorb nitrogen and an increase in tea yield and quality. Secondly, the study found that sheep manure fertilizer was most effective when applied at 50 cm and 70 cm, showing the strongest effect on soil acidification in tea plantations, the highest root activity of tea trees, the highest soil ammonium nitrogen content and the highest yield and quality of tea leaves. Therefore, in the process of using sheep manure fertilizer for fertilization in tea plantations, the location of the main distribution of the root system of different tea tree varieties and different tree ages should be judged, and then reasonable ditching and burying should be carried out to achieve the best results. This study provided an important practical basis for the restoration of acidified tea plantation soil and its fertilizer management.

## Data availability statement

The original contributions presented in the study are included in the article/**Supplementary Material**. Further inquiries can be directed to the corresponding author.

## Author contributions

XJ and YW: Conceptualization, Visualization, Methodology, Writing – original draft, Formal analysis, Writing – review and editing, Funding acquisition. QZ and SL: Formal analysis, Writing – review and editing. YZ and MD: Methodology, Investigation, Writing – original draft. MC, JY and ZW: Methodology, Investigation. HW: Conceptualization, Visualization, Methodology, Writing – original draft, Formal analysis, Writing – review and editing, Funding acquisition. All authors contributed to the article and approved the submitted version.
