# Supplementary material for: Reasonable deep application of sheep manure fertilizer to alleviate soil acidification to improve tea yield and quality
Source: Front Plant Sci. 2023 Jun 23;14:1179960. doi: 10.3389/fpls.2023.1179960 (PMC10327554; doi:10.3389/fpls.2023.1179960)
Supplement: Supplementary file 1 [file DataSheet_1.pdf]

Supplementary Materials

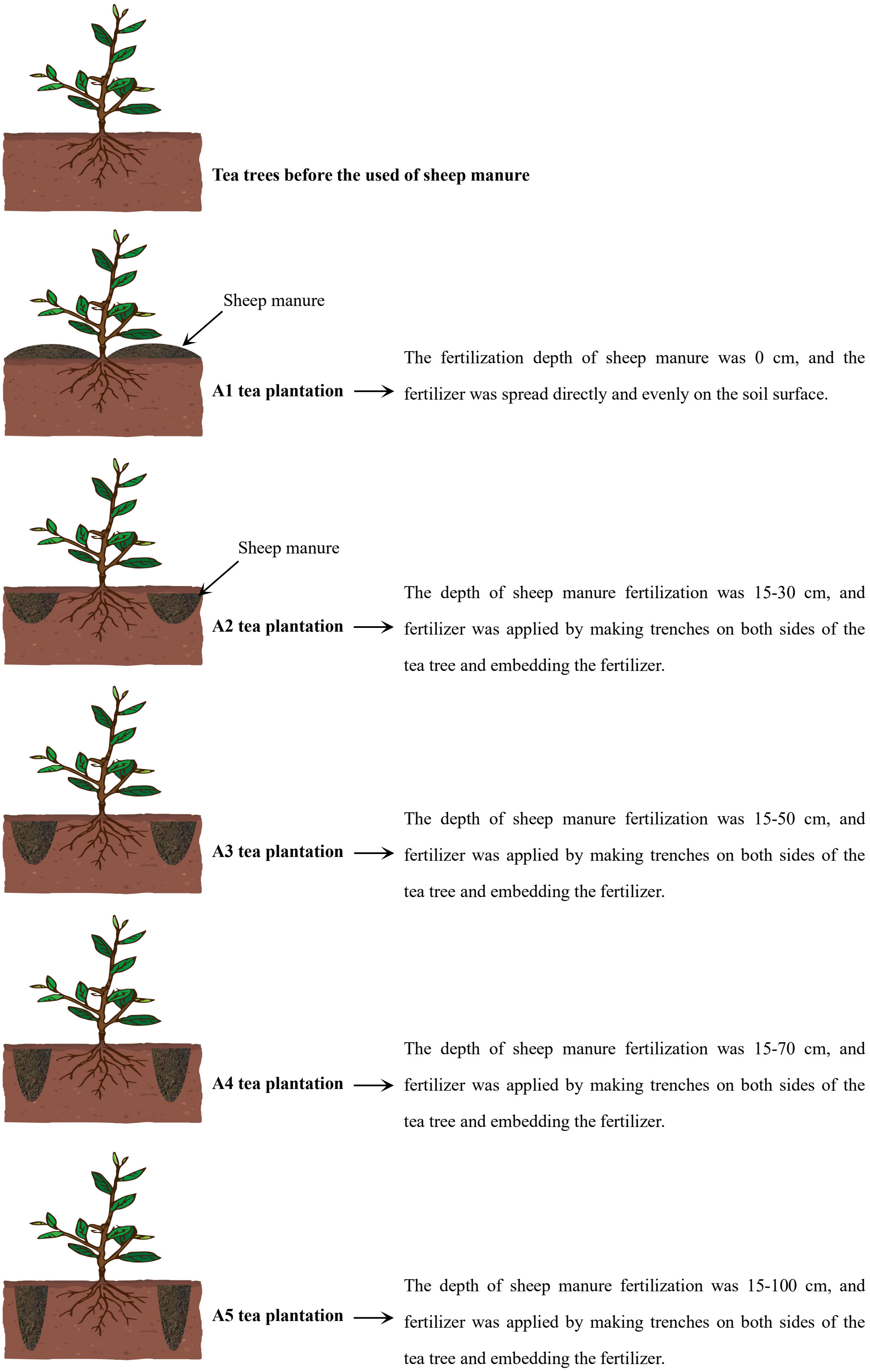

Fig. S1. Schematic diagram of tea plantations with different fertilization depths of sheep manure

Table S1 Indexes of tea tree and soil in the experimental tea plantation in May 2017

	Sample number	Numerical range	Average	Standard deviation	Coefficient of variation (%)
pH value	61	3.09 ~ 3.28	3.15	0.11	3.49
Yield (kg/hm^2)	61	3104 ~ 3365	3186	187	5.87
Amino acid (mg/g)	61	13.02 ~ 13.49	13.24	0.41	3.10
Theanine (mg/g)	61	5.51 ~ 6.08	5.86	0.26	4.44
Root activity (μg/mL·g·h)	61	2.97 ~ 3.41	3.19	0.23	7.21
Total root nitrogen content (mg/kg)	61	0.25 ~ 0.27	0.26	0.02	7.69
Nitrate nitrogen (mg/kg)	61	54.98 ~ 57.75	56.58	1.18	2.09
Ammonium nitrogen (mg/kg)	61	0.91 ~ 1.06	0.98	0.12	12.24
Nitrifying bacteria(10^5 cfu/g·soil)	61	19.97 ~ 20.65	20.36	0.43	2.11
Ammonifying bacteria(10^7 cfu/g·soil)	61	8.06 ~ 8.74	8.37	0.48	5.73
Nitrification intensity (%)	61	32.27 ~ 33.24	32.86	0.51	1.55
Ammoniation strength (mg/100 mL)	61	16.85 ~ 18.13	17.14	1.16	6.77
Urease (μmol/min·L)	61	122.55 ~ 127.49	124.51	3.86	3.10
Protease (μmol/min·L)	61	8.84 ~ 9.82	9.25	0.63	6.81
Nitrate reductase (μmol/min·L)	61	8.25 ~ 8.73	8.48	0.31	3.66
Nitrite reductase (μmol/min·L)	61	163.05 ~ 167.81	165.76	2.75	1.66
nifH gene (×10^6 Copy number/g·soil)	61	2.12 ~ 2.39	2.26	0.18	7.96
amoA-AOA gene (×10^6 Copy number/g·soil)	61	676.53 ~ 697.28	686.54	12.36	1.80
nirK gene (×10^6 Copy number/g·soil)	61	42.52 ~ 44.29	43.18	1.38	3.20
nirS gene (×10^6 Copy number/g·soil)	61	38.12 ~ 39.96	38.95	1.12	2.88
narG gene (×10^6 Copy number/g·soil)	61	3.13 ~ 3.35	3.26	0.12	3.68
nosZ gene (×10^6 Copy number/g·soil)	61	41.68 ~ 43.36	42.56	1.14	2.68
